# Dengue infection in mice inoculated by the intracerebral route: neuropathological effects and identification of target cells for virus replication

**DOI:** 10.1038/s41598-019-54474-7

**Published:** 2019-11-29

**Authors:** J. F. S. Amorim, A. S. Azevedo, S. M. Costa, G. F. Trindade, C. A. Basílio-de-Oliveira, A. J. S. Gonçalves, N. G. Salomão, K. Rabelo, R. Amaral, L. H. M. Geraldo, F. R. S. Lima, R. Mohana-Borges, M. V. Paes, A. M. B. Alves

**Affiliations:** 1 Laboratory of Biotechnology and Physiology of Viral Infections, Oswaldo Cruz Institute, Oswaldo Cruz Foundation, Rio de Janeiro, Brazil; 2 Laboratory of Virological Technology, Institute of Technology in Immunobiology (Bio-Manguinhos), Oswaldo Cruz Foundation, Rio de Janeiro, Brazil; 3 Gaffree & Guinle University Hospital, Federal University of the State of Rio de Janeiro (UNIRIO), Rio de Janeiro, Brazil; 4 Interdisciplinary Laboratory of Medical Research, Institute Oswaldo Cruz, Oswaldo Cruz Foundation, Rio de Janeiro, Brazil; 5 Laboratory of Glial Cell Biology, Institute of Biomedical Sciences, Federal University of Rio de Janeiro, Rio de Janeiro, Brazil; 6 Laboratory of Structural Genomics, Institute of Biophysics Carlos Chagas Filho, Federal University of Rio de Janeiro, Rio de Janeiro, RJ Brazil

**Keywords:** Neuroscience, Dengue virus

## Abstract

Dengue is an important arboviral infection, causing a broad range symptom that varies from life-threatening mild illness to severe clinical manifestations. Recent studies reported the impairment of the central nervous system (CNS) after dengue infection, a characteristic previously considered as atypical and underreported. However, little is known about the neuropathology associated to dengue. Since animal models are important tools for helping to understand the dengue pathogenesis, including neurological damages, the aim of this work was to investigate the effects of intracerebral inoculation of a neuroadapted dengue serotype 2 virus (DENV2) in immunocompetent BALB/c mice, mimicking some aspects of the viral encephalitis. Mice presented neurological morbidity after the 7^th^ day post infection. At the same time, histopathological analysis revealed that DENV2 led to damages in the CNS, such as hemorrhage, reactive gliosis, hyperplastic and hypertrophied microglia, astrocyte proliferation, Purkinje neurons retraction and cellular infiltration around vessels in the pia mater and in neuropil. Viral tropism and replication were detected in resident cells of the brain and cerebellum, such as neurons, astrocyte, microglia and oligodendrocytes. Results suggest that this classical mice model might be useful for analyzing the neurotropic effect of DENV with similarities to what occurs in human.

## Introduction

Dengue is one of the most important diseases caused by an arbovirus, the dengue virus (DENV), which affects 96 million people annually worldwide, with 396 million estimated infections^1^. The disease has a broad range manifestation, varying from a life-threatening mild flu-like illness, known as dengue fever, to severe dengue^2^. The virus belongs to the family *Flaviviridae*, genus *Flavivirus*, and consists of four antigenically distinct serotypes (DENV 1–4)^3^.

Although DENV is classically characterized as a non-neurotropic virus, in the last decades several studies led to a different understanding of the clinical profile of the dengue disease. In addition to a variety of non-specific signs and symptoms, the disease may also present manifestations including neurological involvements^4^. The emergence of neurological signs in human patients infected with DENV was first reported in 1976^5^. Nowadays, encephalitis and encephalopathy stand out as the most common neurological signs resulting from DENV infection^6^.

Several intrinsic and extrinsic factors of the host and the virus, such as age, ethnicity, presence of chronic diseases, specific DENV genotypes, sequential infections with different serotypes and the period between these episodes may be related to the development of severe dengue cases, and consequently for occurrence of neurological manifestations^7,8^. Involvement of the central nervous system (CNS) in patients with dengue may also be the result of a non-specific and secondary complication of the disease, characterized by a metabolic disorder (encephalopathy) or autoimmune reaction, such as disseminated acute encephalomyelitis, optic neuromyelitis, optic neuritis, myelitis and Guillain- Barré syndrome^9–13^. Some studies also indicate a direct action of the virus, including encephalitis, meningitis and myelitis, with DENV invasion in the nervous tissue^14,15^.

In recent years, the incidence rate of neurological signs in dengue patients ranged from approximately 0.5 to 20%, and the virus has been identified in 4–47% individuals with encephalitis or with suspected infection in the CNS^4,16^. Besides, the virus or DENV antigens have been detected in the brain tissue and cerebrospinal fluid (CSF) from several dengue fatal cases^17–19^. In fact, the CNS commitment in the dengue disease has been reported in 25 countries covering almost all continents, affecting individuals aged from 3 months to 60 years^20^. However, the actual number of confirmed dengue cases that are associated with neurological manifestations is underestimated due to the oligosymptomatic nature of the disease and the limited number of studies on the pathogenesis of cases considered as atypical.

On the other hand, the use of animal models is an important tool for better understanding the pathogenesis of dengue, including the induction of neurological changes. Immunocompetent mice may be an interesting model, since the integrity of the immune system is maintained, which may influence the pathogenesis of the disease, in addition to its relevance in studies for vaccine development. However, immunocompetent mice are generally resistant to DENV-induced disease^21^. Alternatively, approaches using neuroadapted viral samples and/or inoculation with high doses of DENV may be useful for the development of experimental models and evaluation of neuropathology^22–24^. Besides, inoculation by the intracerebral (i.c.) route of neuroadapted virus, which induces neurological clinical signs and can lead to animal death, may be a robust tool to investigate the involvement of the central nervous system in the pathogenesis of dengue^24^.

Hence, in the present work we aimed to investigate the effect of the i.c. inoculation of a neuroadapted DENV2, strain  New Guinea C (NGC), in BALB/c mice, which mimics some aspects of the viral encephalitis. This mouse model was previously used to test different vaccines against dengue^25–28^. Animals inoculated with the DENV2 presented morbidity, mainly hind leg paralysis and commitment of spinal cord, after the 7^th^ day post infection. Histopathological analysis showed that virus inoculation led to tissue damage in the CNS of mice infected, such as hemorrhage, reactive gliosis, hyperplastic and hypertrophied microglia, astrocyte proliferation (gliosis reaction), Purkinje neurons retraction and infiltrates around vessel in the pia mater and in neuropil. Virus replication was ascertained by real time PCR, as well as by detection of the non-structural 3 (NS3) protein of DENV2, which revealed virus tropism and replication in neural cells, such as neurons and neuroglial cells (astrocytes, microglia and oligodendrocytes).

## Results

### Dye inoculation (Nankin) in the nervous system of BALB/c mice

**Figure 1:**
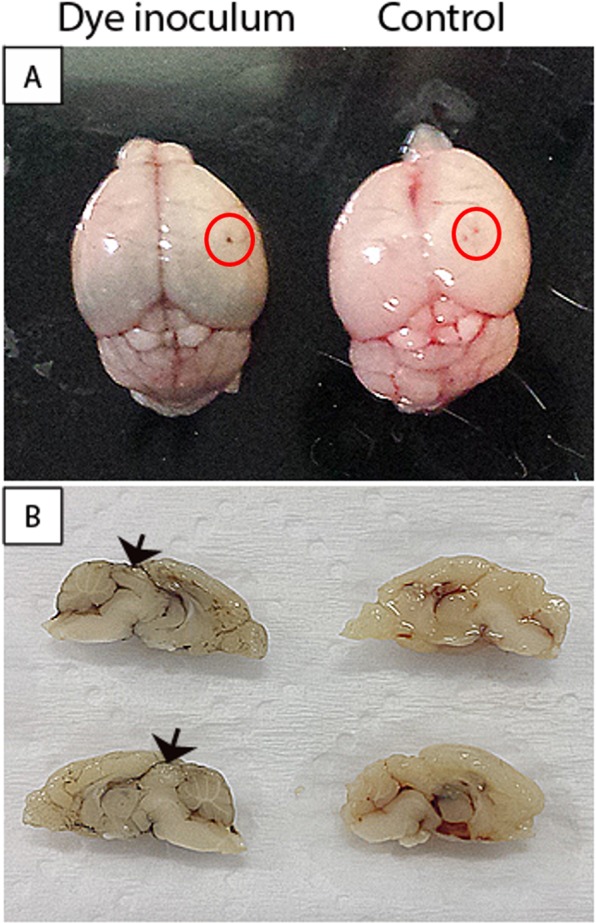
Macroscopy of the brain/cerebellum of BALB/c mice injected with Nankin dye by the intracranial route. Brain and cerebellum inoculated with Nankin dye (left of figures) or PBS (right of figures). The inoculation site is shown inside red circles in upper panel (**A**) and the brain and cerebellum meridian section is exposed in bottom panel (**B**). Black arrowheads indicate the Nankin dye.

In order to verify the site of the DENV inoculation in CNS of BALB/c mice, animals were injected with Nankin dye, simulating the virus inoculum, or with PBS as control. Macroscopic analysis in the brain and cerebellum of Nankin injected animal revealed a darker coloration in these tissues compared to control animal. The presence of dye in main vessels (Fig. 1A,B) indicated dispersion of the inoculum to the bloodstream, which probably also occurs after DENV inoculation by the same route. As expected, control mouse presented brain and cerebellum with normal appearance, with lighter and rosy color due to the presence of blood in the vessels (Fig. 1A,B).

### Clinical manifestation of DENV infection in BALB/c mice inoculated by the intracerebral route

**Figure 2:**
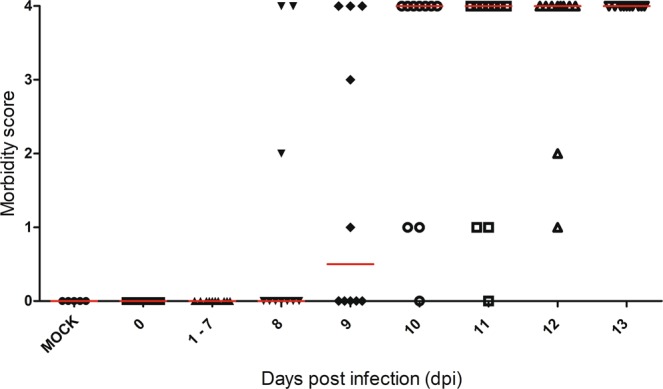
Morbidity and mortality of BALB/c mice inoculated with NGC DENV2. BALB/c mice were inoculated with the neuroadapted DENV2 by the i.c. route. Mock control group were injected with noninfected culture cell supernatant. Mortality and clinical signs of infection were monitored during 13 dpi. Morbidity score and mortality were noted according to an arbitrary scale ranging from 0 to 4 (0 = absence of morbidity, 1 = mild paralyses in one hind leg or alteration of the spinal column with small bump, 2 = one severe hind leg paralyses and alteration of the spinal column with small bump or two severe hind leg paralyses, 3 = two severe hind leg paralyses and bump deformed spinal column and 4 = death). Red line indicates the median of mouse morbidity in each timepoint of infection.

Morbidity and mortality were monitored in DENV-2 infected animals from 1 day to 13 days post infection (dpi). Morbidity degrees were evaluated in each animal according to severity, assigned as 0 (absence of morbidity) to 3 (severe paralyses in hind legs and deformed spinal column) and 4 as death. In general, the strength in animal legs started to decrease after the 7^th^ dpi and all DENV2-inoculated mice died between the 8^th^ and 13^th^ day after infection (Fig. 2). Half of animals presented high morbidity degree with commitment of the CNS, mainly hind leg paralysis and alteration of spinal cord, preceding death. However, the remaining animals died without having previous morbidity. As expected, all mock-injected animals survived without morbidity (Fig. 2).

### Histological changes in the brain tissue of BALB/c mice infected with DENV2

**Figure 3:**
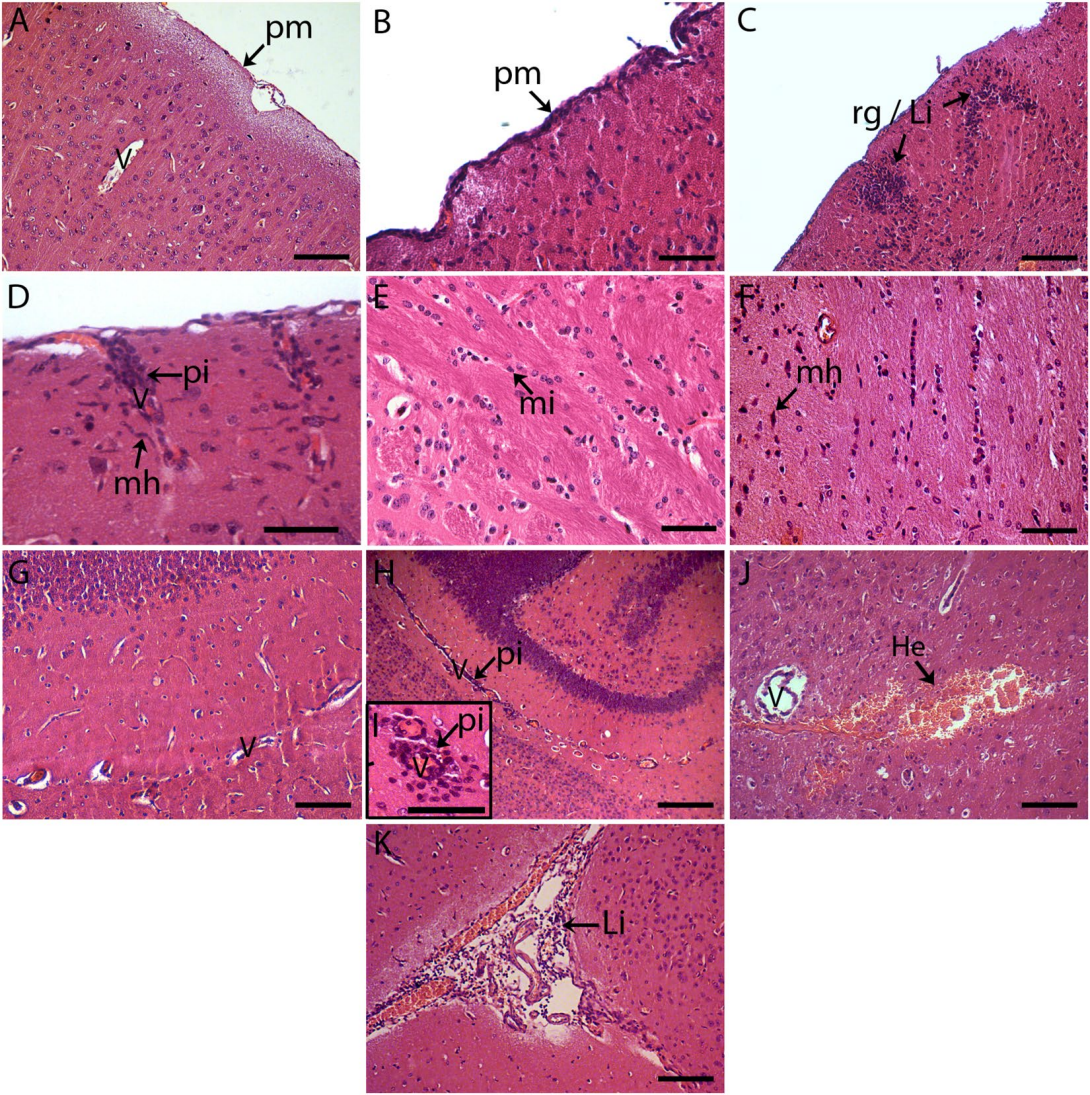
Histopathological aspects of brain tissue in BALB/c mice infected with DENV2 or in non-infected animals (mock). Brain cortex of mice inoculated with mock (**A,E,G**) or with DENV2 (**B–D,F,H–K**). In DENV2 infected animals, we observed: inflammatory infiltrates in the pia mater (**B**), around vessels (**D,H,I**) and in the choroid plexus (**K**); cellular cortex with foci suggesting reactional gliosis with lymphocytic infiltrates (**C**); microglial hyperplasia in cellular cortex and white matter (**D,F**); and hemorrhage areas (**J**). He – hemorrhage; Li- lymphocytic infiltrate; mi – microglia; mh- microglial hyperplasia; pi – perivascular infiltrate; pm - pia-mater; rg - reactive gliosis; V – vessel. Sections were stained with H.E. and visualized under light field microscopy. Bars (I) = 10 μm; (A,C,G,J,K) = 50 μm; (B,D,E,F) = 25 μm; (H) = 100 μm.

We then investigated the histopathology of the brain tissue, since mice were inoculated with the virus by the i.c. route and several animals presented clinical manifestations related to CNS disorders and succumbed to the disease. The analysis was performed by comparing mouse groups inoculated with mock or with DENV2, and tissues were collected at different time points after infection (1, 3, 5, 7 and 8 to 11 dpi). As expected, control animals presented normal cellular aspects and tissue structures in the brain. Neuronal and glial cells, parenchymal cortex and white matter, as well as the cellular lining of the pia mater and blood vessels, showed no abnormalities (Fig. 3A,E,G). On the other hand, histopathological aspects could be noted in the brain tissues of DENV2-inoculated mice throughout of all the kinetics of the infection. Hemorrhages were observed in the cerebral cortex, from the beginning to the end of the infection kinetics (Fig. 3J). The microglial hyperplasia was present in the 1^st^ dpi (Fig. 3F) and inflammatory infiltrates began to appear around 3 dpi in hippocampus area and around vessels (Fig. 3H,I). However, only in the 5^th^ dpi, the lymphocyte infiltrates were detected in choroid plexus inside third ventricle (Fig. 3K). Inflammatory infiltrates in pia mater (Fig. 3B) and perivascular, as well as microglia hyperplasia, significantly increased in cerebral cortex from 7^th^ day to the last days of the kinetics (Fig. 3D). Focal areas of reactive gliosis, accompanied or not by lymphocytic infiltrate, were more apparent after the 7^th^ dpi (Fig. 3C).

**Figure 4:**
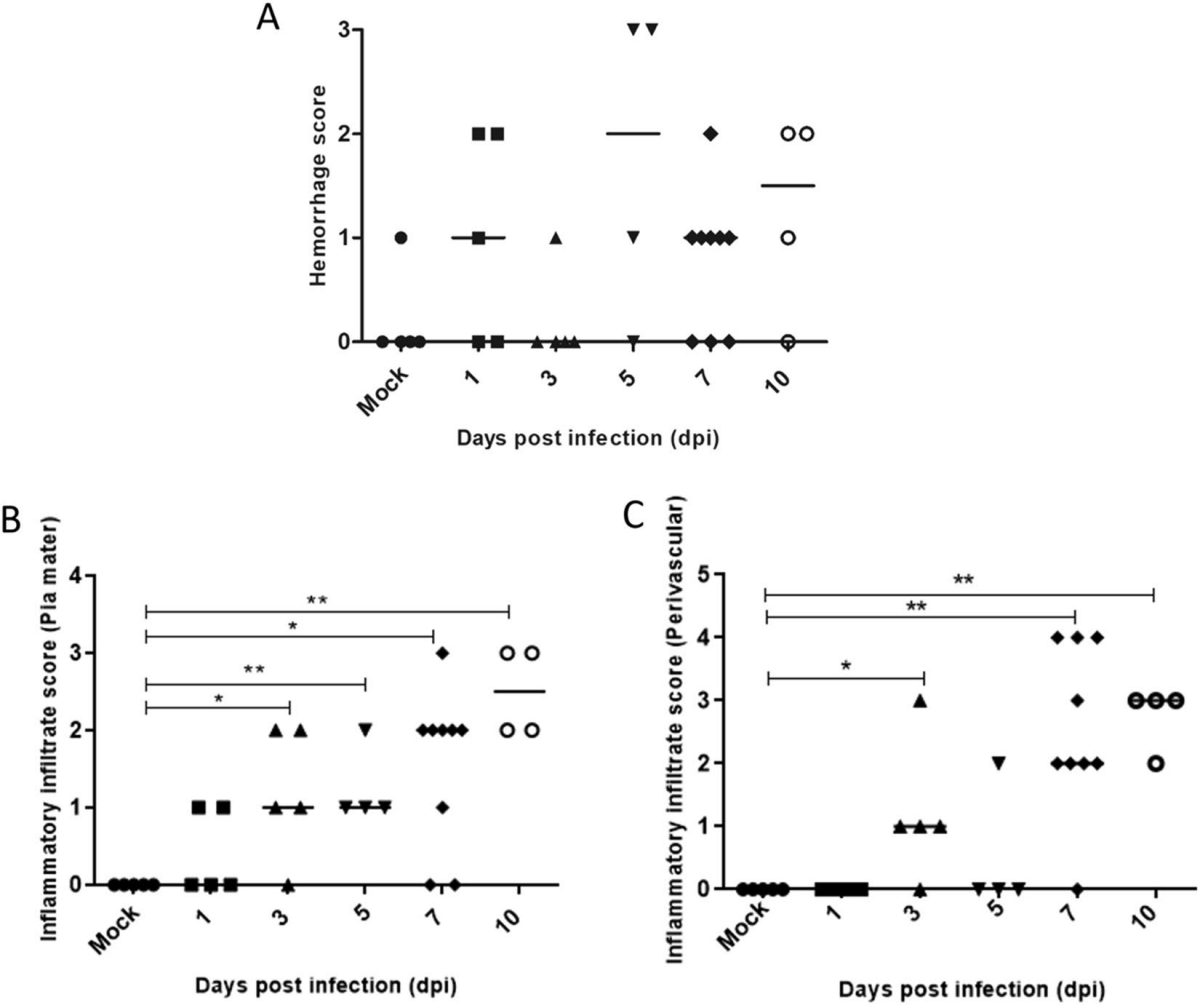
Semiquantitative analysis of alterations in the brain tissue of DENV2-infected mice on days 1^st^, 3^rd^, 5^th^, 7^th^ and 10^th^ dpi. Scores were assigned using a subjective scale from 0 to 4 for the different alterations (n = 4 to 9). Circulatory changes and Inflammatory infiltrates: 0 = no damage, 1 = mild, 2 = moderate, 3 = severe and focal, and 4 = severe and diffuse. Bars = median. Asterisks indicate significant differences using Mann-Whitney statistical test (*p ≤ 0.0185; **p ≤ 0.0050).

Alterations in the brain tissue were measured through a semiquantitative analysis. The histological changes were scored using a 0–4 point scale, in which 0 = no damage, 1 = mild, 2 = moderate, 3 = severe and focal, and 4 = severe and diffuse. Quantification revealed that hemorrhage was more severe on the 5^th^ dpi, but with no statistical significance (Fig. 4A). Evaluation of inflammatory infiltrates, as those found in the pia mater, showed a slight increase in the 3rd dpi and a significant increase from the 7th dpi until the end of the experiment (Fig. 4B). Perivascular infiltrates also showed the same pattern, appearing on the 3^rd^ dpi but only with a significant increase after the 7^th^ dpi (Fig. 4C).

### Histological changes in the cerebellar tissue of BALB/c mice infected with DENV2

**Figure 5:**
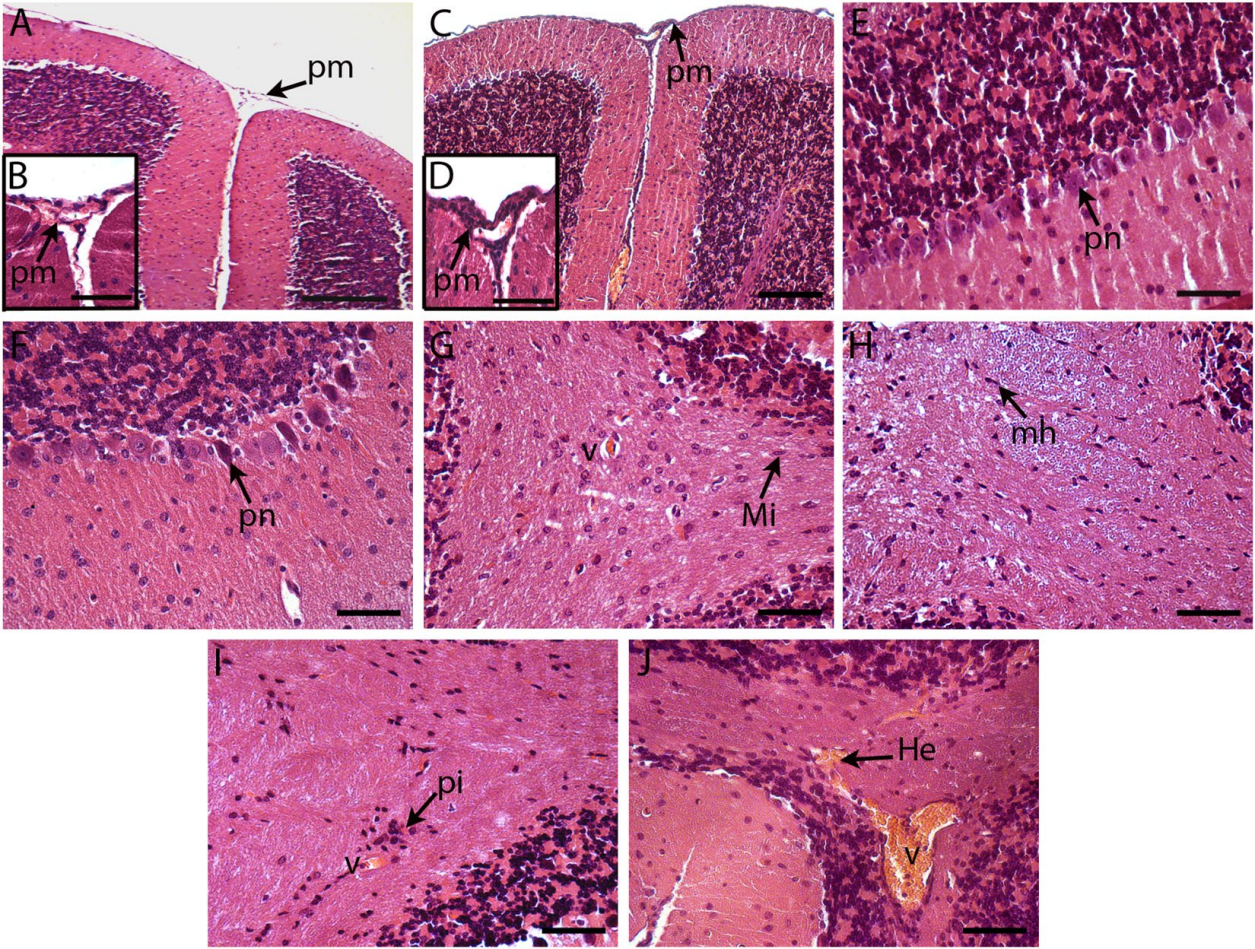
Histopathological aspects of cerebellar tissue in BALB/c mice infected with DENV2 or inoculated with mock. Cerebellar tissue of a mice inoculated with mock (**A,B,E,G**) or with DENV2 (**C,D,F,H–J**). Animals infected with DENV2 exhibiting lymphocytes infiltrates in the pia mater and perivascular (**C,D,I**); acidosis and retraction of Purkinje neurons (**F**); microglia hyperplasia (**H**) and hemorrhagic foci in white matter (**J**). pm – pia mater; pn- Purkinje neuron; Mi – Microglia; mh –microglial hyperplasia; pi - perivascular infiltrate; He – hemorrhage; V – vessel. Sections were stained with HE and visualized under light field microscopy. Bars (A) = 100 μm; (C) = 50 μm; (E,F,G,H,I,J) = 25 μm; (B,D) = 10 μm.

Histopathological analysis was also performed on the cerebellar tissue. Changes caused by DENV2 infection in the cerebellum were similar, but milder than those observed in the brain. Early infection was characterized by microglial hyperplasia in the white matter (Fig. 5H), but this pattern decreases when reaching the end of the kinetics. On the 3^rd^ dpi, an acidosis and morphological change in Purkinje neurons was observed, indicating degeneration of these cells (Fig. 5F). The end of the infection kinetics was marked by discrete lymphocyte infiltrates in the pia mater (Fig. 5C,D) and around the blood vessels in the white matter (Fig. 5I), as well as focal areas of hemorrhage mainly in the white matter (Fig. 5J). As expected, the mock control group did not show relevant changes compared to infected mice (Fig. 5A,B,E,G).

### Glia and microglial cell activation in the brain of BALB/c mice infected with DENV2

**Figure 6:**
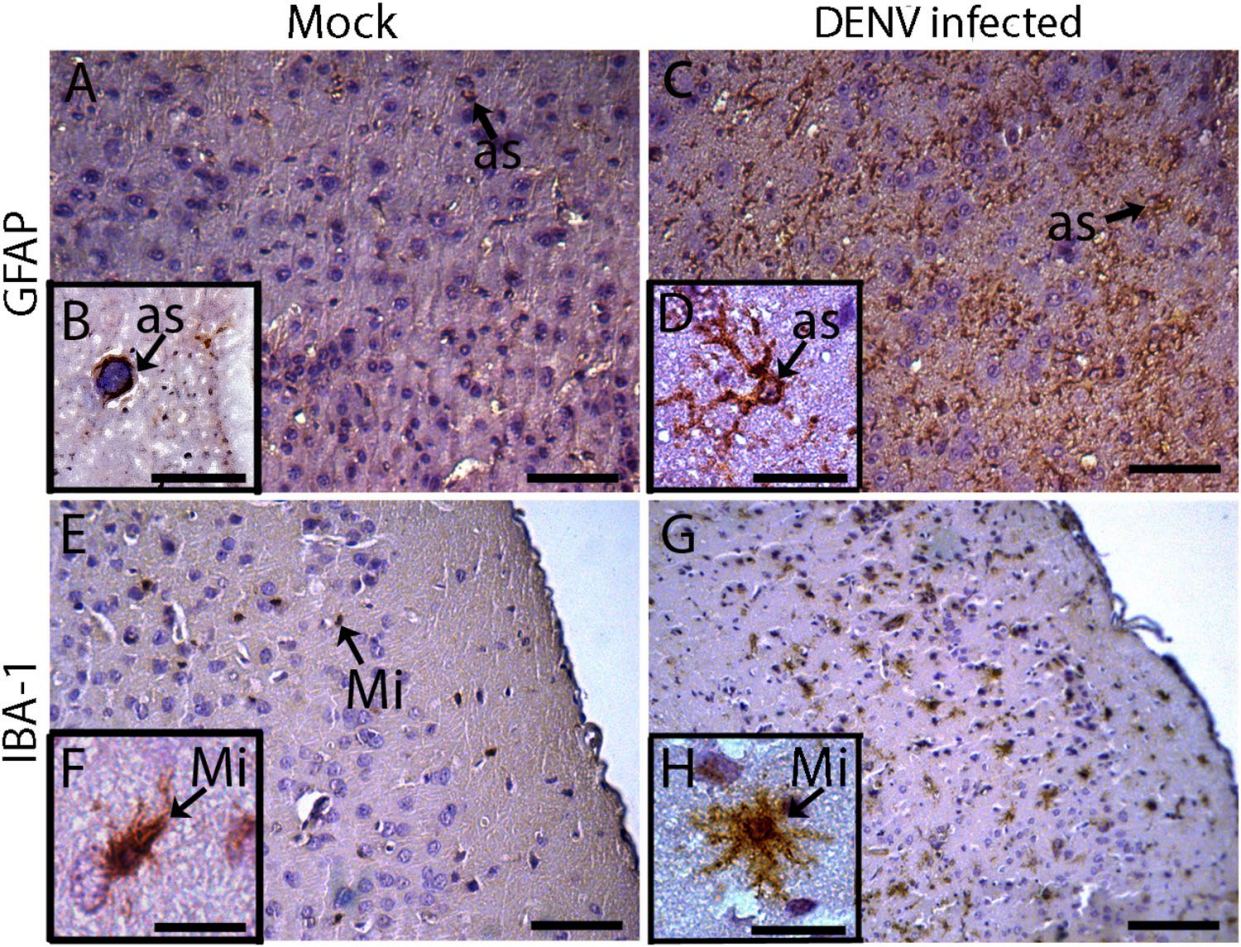
Detection of glial cells in the brain tissue from BALB/c mice infected with DENV2. Tissue samples were collected from animals inoculated with mock (**A,B,E,F**) or with DENV2 (10 dpi) (**C,D,G,H**). Sections were incubated with anti-GFAP (**A–D**) or anti-IBA-1 (**E–H**) antibody, followed by incubation with peroxidase-conjugated rabbit anti-IgG antibody. Detection of astrocytes by the GFAP (**A–D**) and microglia by the IBA-1 protein (**E–H**). The sections were stained with Harris hematoxylin. Mi – microglia; as – Astrocyte. Bars (B,D,F,H) = 10 μm; (A,C,E) = 25 μm; (G) = 50 μm.

After the histopathological findings in the cerebral tissue, revealing the increase and activation of glial cells mainly in the 10^th^ dpi, immunohistochemical assays were performed for detection of neuroglial cells, such as astrocyte and microglia, with tissue samples obtained at this time point of infection. Anti-GFAP and anti-IBA antibodies were used to detected astrocyte and microglial cells, respectively, in samples collected from infected and mock control animals. Comparative analysis between brain tissues from infected and control mice, revealed a remarkable increase of cells labeled for GFAP (Fig. 6C,D) and IBA (Fig. 6G,H) in the cerebral cortex of DENV2-inoculated animals. Hypertrophy of these cells was also noted. In contrast, no such alterations were detected in control animals (Fig. 6A,B,E,F).

### Quantification of DENV2 RNA in the brain of infected BALB/c mice

**Figure 7:**
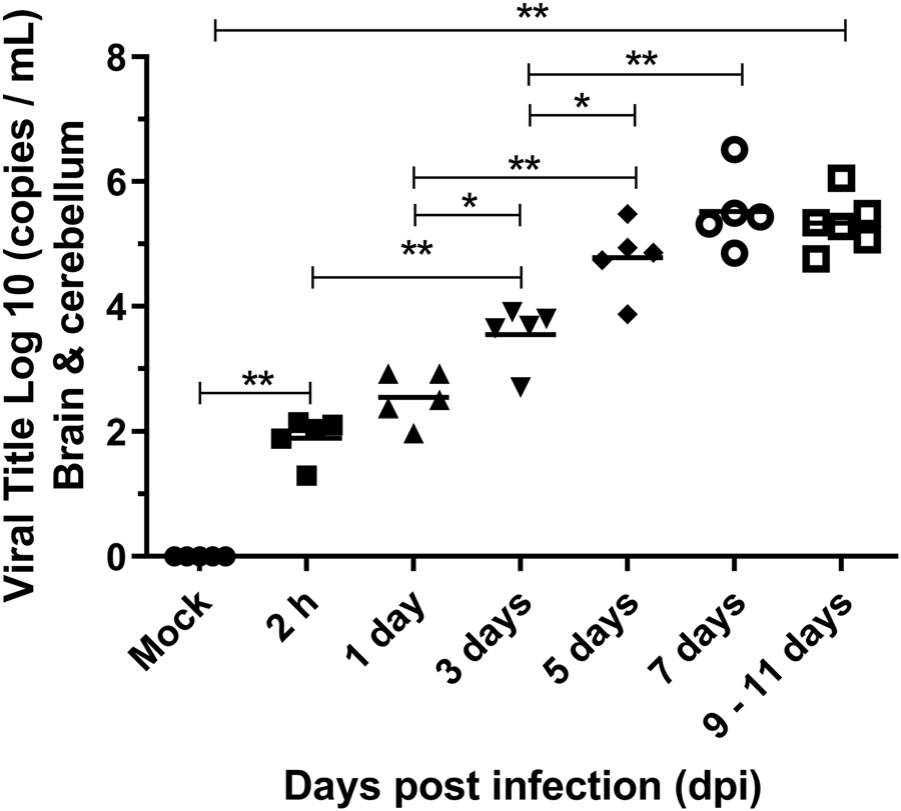
Quantification of viral RNA copies by RTq-PCR in the brain and cerebellum of BALB/c mice infected with DENV2. The DENV2 RNA was measured by real-time RT-PCR using primers annealing to a conserved region of the dengue envelope gene. Viral load in animals inoculated with DENV2 was assessed 2 h to 9–11 dpi or in mock controls (n = 5 or 6). Values are expressed in Log 10. Bars = median. Asterisks indicate significant differences using Mann-Whitney statistical test (*p ≤ 0.03; **p ≤ 0.001).

Quantification of the total viral RNA was performed by RT-qPCR in pooled samples from the brain and cerebellum of infected mice, in order to investigate the presence of DENV2 on the CNS. Samples were collected 2 h, 1, 3, 5, 7 and 9–11 days after the DENV2 inoculation while control samples were obtained 3 days after mock injection. As expected, no viral RNA was detected in samples collected from control mice. On the other hand, the DENV RNA was detected 2 h post infection and viral load increased significantly in the following days, reaching maximum at the endpoint of the experiment (7 and 9–11 dpi) (Fig. 7).

### Detection of viral antigen (NS3) in the cerebral and cerebellar tissue of infected mice

**Figure 8:**
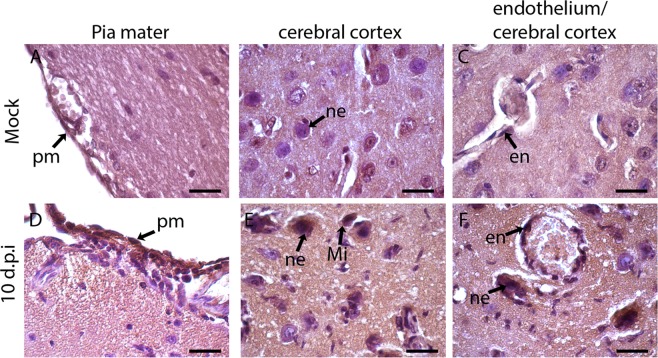
Detection of NS3 protein in the brain tissue of DENV2-infected BALB/c mice. The cerebral sections were incubated with polyclonal anti-NS3 antibodies and with peroxidase conjugated anti-rabbit IgG. Mice inoculated with mock (**A–C**) or with DENV2 (**D,E**). Detection of NS3 protein in neurons, endothelial cells, cells lining the pia mater and microglia from animals infected with DENV2 on the 10^th^ dpi (**D–F**). Sections were stained with Harris Hematoxylin. pm - pia mater; Mi - microglia; ne - neuron; en - endothelium. Bars = 10 μm.

**Figure 9:**
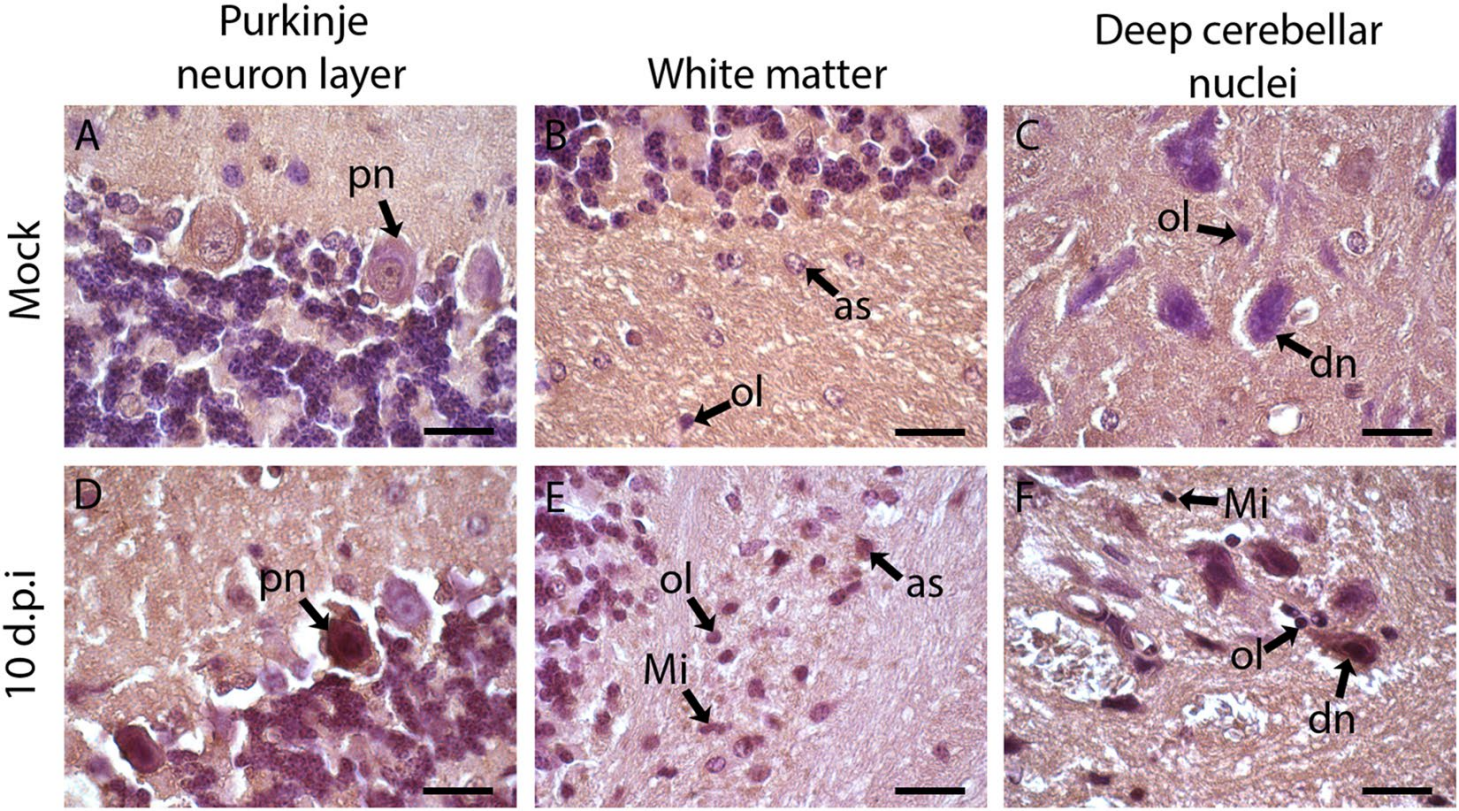
Detection of NS3 protein in the cerebellar tissue of BALB/c mice infected with DENV2. The cerebellar sections were incubated with polyclonal anti-NS3 antibodies and with peroxidase conjugated anti-rabbit IgG. Animals inoculated with mock (**A–C**) or with DENV2 (**D–F**). Detection of NS3 protein in purkinje neuron and in oligodendrocytes, astrocytes, microglia in the white matter and neurons in the deep cerebellar nuclei on the 10 dpi. (**D–F**). Sections were stained with Harris Hematoxylin. Mi - microglia; pn – purkinje neuron; dn- deep cerebellar nuclei neurons, ol – oligodendrocytes, as - astrocytes. Bars = 10 μm.

An immunohistochemical assay for detection of the DENV NS3 protein was performed in the brain tissue of infected mice in order to investigate virus replication. Tissue samples were collected 10 dpi, since the maximum DENV2 load, detected by RT-PCR in the brain, was observed after the 7^th^ dpi. The mock-injected mouse tissue was used as negative control and, as expected, no NS3 was detected in this group (Fig. 8A–C). In contrast, positive reaction was observed in the cerebral tissue of DENV2-infected animals revealing NS3 labeling in neuronal cells, cells that line the pia mater, endothelial cells and microglia (Fig. 8D–F). The presence of the DENV NS3 antigen was also investigated in cells of the cerebellar tissue. As observed in the brain tissue, no NS3 was detected in the mock control group (Fig. 9A–C). Tissue samples from infected mice, in turn, showed the presence of the NS3 protein in nerve cells of the cerebellar tissues, such as Purkinje neurons (Fig. 9D), microglia in the white matter (Fig. 9E) and neurons in the deep cerebellum nuclei (Fig. 9F) in the 10^th^ dpi. Therefore, results suggest virus replication in nerve cells in both the brain and cerebellum of infected mice.

### Detection of virus proteins in primary neuronal culture cells infected with DENV2

**Figure 10:**
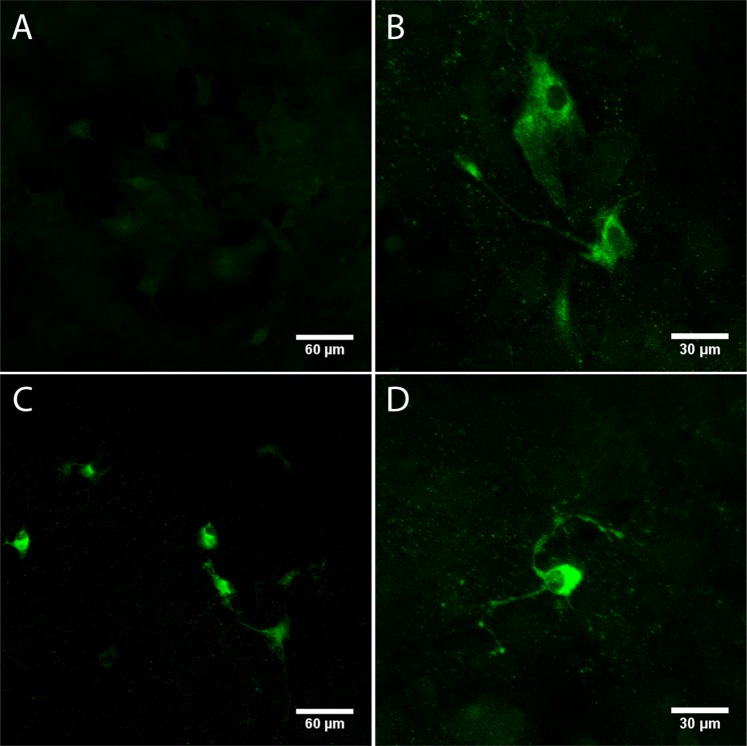
Detection of the NS3 protein in primary culture of neuronal cells infected with DENV2. Cell cultures were incubated with anti-NS3 antibody and FITC conjugated anti-rabbit IgG, 48 h post-infection. Cells incubated with mock (**A**) or DENV2 (**B–D**); Detection of NS3 protein in neurons (**B**), microglia (scattered and pyramidal format in the lower right corner of the image) (**C**) and astrocytes (rounded format and apparent nucleus) (**C,D**).

**Figure 11:**
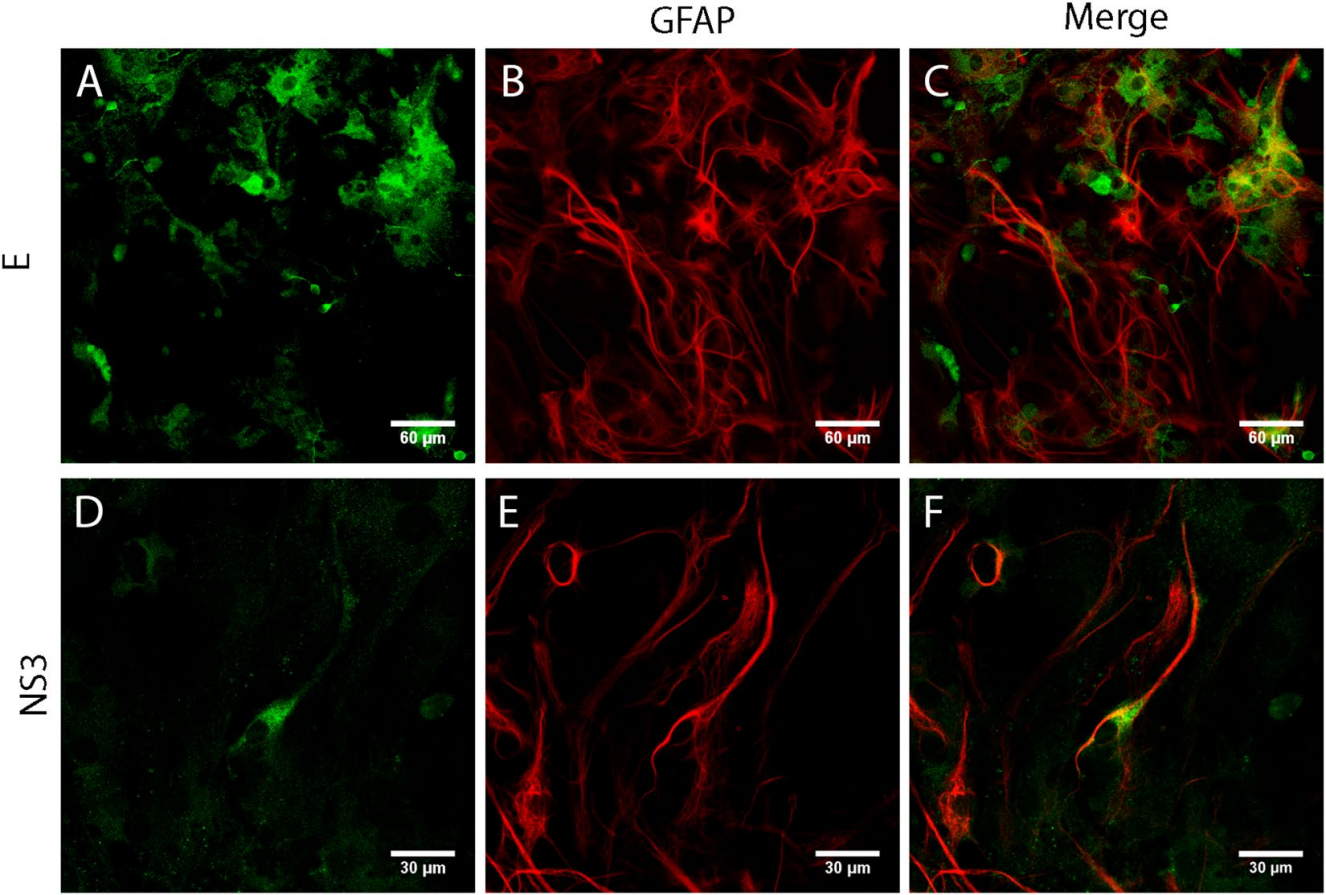
Primary culture of astrocytes infected with DENV2. Cells were incubated with anti-GFAP TRITC-conjugated (**B,E**) and anti-E (**A**) or anti-NS3 (**D**) polyclonal antibodies plus anti-rabbit FITC-conjugated IgG. Merge: Co-stain of anti-GFAP and anti-E (**C**) or anti-NS3 (**F**).

The neurotropism of the DENV2 NGC strain used for inoculation of adult immunocompetent BALB/c mice was also investigated in primary neuronal culture cells. Cells were obtained from the brain and cerebellum of naïve newborn BALB/c mice and were infected for 48 h with the DENV2. Virus replication was evidenced by immunofluorescence assay for detection of the DENV NS3 protein in a monolayer of mixed nerve cells. As indicated in Fig. 10, the NS3 protein was observed in neurons (Fig. 10B), microglial (Fig. 10C) and astrocyte (Fig. 10C,D) cells. Since there were no reports in literature about DENV infection in astrocytes, the mixed neuronal cell monolayer infected with DENV2 were also co-stained for GFAP and for NS3 or E viral proteins in order to confirm the virus tropism for these cells. Results revealed co-localization of GFAP and the DENV proteins, confirming the virus presence and replication in astrocytes (Fig. 11).

## Discussion

The lack of an animal model mimicking all the aspects of the dengue disease observed in humans is a strong limitation to understand the DENV pathogenesis. Mice are generally used in initial testing of vaccine prototypes against different flaviviruses, while non-human primates are only used in later studies prior to clinical trials, due to ethic concerns and cost. Immunocompetent mice present natural resistance to DENV infection, mainly because the virus is not able to subvert the mouse IFN-α/β antiviral response as it happens in humans^29,30^. One alternative is the use of immunocompromise mice, in particular those deficient in the IFN-α/β and IFN-γ pathway, which develop clinical signs after infection with DENV similar to those observed in humans^21^. However, this mouse model presents limitations, since it is known that innate and adaptive responses have strong impact on dengue infection, and their use for vaccine tests is controversial^31,32^. Immunocompetent mice, in turn, are still an alternative, in particular with the use of neuroadapted viral samples. In fact, the most traditional immunocompetent mouse model for testing vaccines against DENV is the use of a brain-adapted virus inoculated by the intracerebral route, which provides a straightforward readout parameter because the infection is usually lethal^25,27,33^. Albeit protection in these animals is normally reduced to evaluation of morbidity and/or survival rates after virus challenge, we have recently shown the systemic effect of the DENV2 inoculation in BALB/c mice by the i.c. route.^24^. Yet, the direct effect in the brain of animals inoculated with this virus is still uncertain.

In this regard, the present work aimed to contribute for elucidation of the neuropathology of dengue, by evaluating the effects of the DENV2 infection in brain and cerebellar tissues of BALB/c mice inoculated by the intracerebral route with the New Guinea C DENV2 strain. The NGC strain was originally isolated from infected human followed by serial passages in newborn and young adult brain mice^34^. The neuroadaptation could be confirmed not only by morbidity and mortality caused by the DENV infection but also by the number of infective virus particles used in the inoculum. In fact, our previous virus stock with less mouse brain passages lead to approximately 4 LD_50_ with an inoculum of 4.32log_10_ PFU^35^, while after one more passage virus infection achieved 40 LD_50_ with an inoculum of only 2.04log_10_ PFU. We, therefore, used this stock for the evaluation of brain damages in adult mice.

Although the intracerebral is not the natural route of the DENV infection, the number of human dengue case with brain commitment has increased in the past years, with neurological signs and symptoms ranging from muscular dysfunction to encephalitis and syndromes, thus indicating the importance of studying the involvement of the CNS during the virus infection^6,36–39^. We observed morphological alterations of the microglia and astrocytes in the nervous tissue of infected animals, and a correlation with the detection of DENV antigens and virus replication. The presence of DENV RNA and antigens has also been observed in CNS and cerebrospinal fluid from several fatal cases^19,40,41^. Besides, the involvement of the CNS was also noticed in mice inoculated by the peripheral route with non-neuroadapted dengue strains, leading to neurological symptoms, such as paralysis or breakdown of the blood-brain barrier with virus detection in the brain of infected animals, thus confirming the DENV neurotropism^42–44^. The DENV neurotropism may be influenced by the virus strain, with more or less affinity for the CNS^45,46^, which brings it closer to other flaviviruses that cause viral encephalitis, such as the St. Louis encephalitis, Japanese encephalitis, West Nile, and Zika virus. Our work evidenced that the DENV2 NGC was able to infect and replicate in the brain and cerebellar tissue, with maximum viral load in the endpoint of the experiments and concomitant histopathological alterations in the cerebral parenchyma. The number of virus RNA copies increased up to the 7^th^ dpi and remained similar at the following points, which may be a consequence of a decrease in virus replication and/or the spread of the virus into the circulation. In fact, we have previously shown the presence of infective virus particles in blood samples of DENV i.c. inoculated animals, mainly at the endpoints of infection, which indicates a systemic dispersion of the virus^24^. In the present work, viral replication was observed in cells from the nervous system, both *in vivo* and *in vitro*. The DENV NS3 antigen, which indicates viral replication, was detected in the infiltrate of the pia mater, as well as in endothelial cells, microglia, neurons, oligodendrocytes and astrocytes in the end of the infection kinetics. As far as we know this is the first report indicating the presence of DENV in leukocyte infiltration into the pia mater.

The clinical signs of infection with commitment of the CNS generally appear after the 7^th^ dpi, when the vertebral column is impaired (curved) and loss of strength or paralysis is observed in the lower and upper limbs. Coincidentally with the onset of morbidity, analyses of the cerebellar and brain tissue have shown that inoculation of DENV2 in BALB/c mice caused pronounced encephalitis and meningitis after the 7^th^ dpi. The brain tissue exhibited thickening of the pia mater with the presence of inflammation and vascular lesions at all points (from 1 to 10 dpi). Animals displayed exacerbation of inflammatory response, with diffuse infiltrates in the cortex and perivascular regions, and reactional gliosis at the end points of the infection kinetics, suggesting a disruption of the blood-brain barrier that led to the emergence of clinical neurological signs present in infected mice. At the same time, the cerebellar tissue also showed inflammation causing thickening of the pia mater and perivascular infiltrates, although this inflammation was milder. Microglial activation and astrogliosis was also reported in mice infected with other flavivirus, such as ZIKV^47^.

In response to neuroinflammation, microglia hyperplasia in cerebral and cerebellar tissues was prominent in the white matter at the beginning of the infection, which was different from what we observed in the cerebral cortex, with more detectable effects at the endpoint of infection. According to other authors, this scenario may occur due to the existence of heterogeneous populations of microglia in different regions of the brain and cerebellum^48,49^. Although the functions of these populations in the CNS have not been deeply investigated, it is known that microglia residing in the white matter of the cerebrum and cerebellum presents a differential immunological responsiveness and, when necessary, it turns over rapidly with a robust innate and adaptive immune response to control tissue homeostasis^50–52^. This characteristic may explain the early activation of microglial populations in the white matter. As a mild inflammation was detected in the cerebellum, microglia population was probably regulated and maintained under resting conditions in the following days of infection, in order to avoid cellular hyperactivation and consequent damages to the tissue environment^48^. On the other hand, since the cerebral cortex shows late inflammation of a greater magnitude and slower response of the cortical microglia^51^, the microglia population may have been expanded and activated late to contain inflammation due to DENV infection.

The morphological identification in our histopathological analysis revealed that most of the inflammatory infiltrate in brain and cerebellum observed at the endpoints of infection consisted of lymphocytes. These findings are in agreement to our previous study with characterization by flow cytometry of the infiltrate cells in the brain of inoculated mice, which showed the presence of CD4 ^+^ and CD8 ^+^ T lymphocytes at the endpoint of infection and suggested a correlation between the presence of lymphocytic infiltrates and hemorrhagic lesions^24^. It is important to note that the inflammation present in the cerebral tissue was not due to the intracerebral inoculation by itself, since no such effects were detected in animals injected with mock. Virus replication seems to play an important role in the observed inflammatory process, although the response to the DENV proteins by itself in triggering this process should not be ruled out.

The inflammatory reaction observed in the brain and cerebellum in the end of the experiment may be a consequence of a response of resident cells, such as activated microglia, and also the drainage of antigens to the cervical lymph nodes that would sensitize T lymphocytes. In line with this rationale, recently it was discovered that viral antigens can be drained by the interstitial fluid of the brain by a particular lymphatic system that would lead them to the cervical lymph nodes, which would activate the T cells that infiltrate the CNS by the blood, causing an inflammatory response^53,54^. Moreover, migration of leukocytes to the brain and cerebellum of infected mice, in pia mater and perivascular infiltrates, would also be facilitated by increased expression of adhesion molecules on endothelial cells stimulated by anti-viral cytokines, as already described^55,56^.

Our analyses also revealed focal areas of reactive gliosis and microglial hyperplasia and hypertrophy in the brain tissue in late points of the DENV2 infection. Activation of astrocytes and microglia was confirmed by the hypertrophy and a marked increase in the number of these cells detected by the immunohistochemical assay. Astrocytes are known to be important in supporting and nutrition of neurons, but they also play a role in the immune response of the brain tissue, since these cells can release pro- or anti-inflammatory cytokines and chemokines which, together with microglia, influence the type of the effector T cell response^57,58^. Astrocytes may also act as antigen-presenting cells, which induces T cell proliferation in the brain^57^. Moreover, the reactive gliosis process can be explained by conversion of mature astrocytes into neural progenitors, with the ability to proliferate and differentiate into neurons and/or new astrocytes^59^. Therefore, the reactive gliosis found in the DENV infected animals may also act by attracting the leukocytes to the brain. Besides, astrocytes that compose the foci of reactive gliosis may have a pro-inflammatory profile, by modulating infiltrated T cells for an inflammatory effector immune response. On the other hand, these glial cells may have proliferated in the process to generate new neurons and astrocytes in order to restore the brain tissue. Further studies will be necessary to evaluate such hypothesis. However, since all mice inoculated with our NGC DENV2 stock succumbed to infection, it was not possible to stablish whether resolving inflammation would lead to survival of animals with a glial scar.

Although our mouse model using 40 LD_50_ is far more lethal than what is observed in humans, it can be a robust tool for protection analysis in vaccine and therapeutic drug tests. Besides, our studies using the neuroadapted DENV2, a classical model for testing vaccines in an immunocompetent mice, reinforces the neurotropic characteristic of the virus with correlation to what may occur in humans, and points it as an useful model for studies of the dengue neuropathology.

## Methods

### Animals

Studies with animals were conducted in 8-week-old male BALB/c mice, specific pathogen free (SPF), purchased from the Multidisciplinary Center for Biological Research (CEMIB, Campinas/SP). Experiments related to infection of primary cells demanded 7 newborn BALB/c mice, which were generated by two pregnant females obtained from the Institute of Science and Biology in Biomodels (ICTB/FIOCRUZ, Rio de Janeiro/RJ). All experiments were performed in accordance with the ethical principles established in the Brazilian College of Animal Experimentation and approved by the Committee of Ethical Animal Use from Oswaldo Cruz Institute/ Oswaldo Cruz Foundation (CEUA-IOC/Fiocruz) (L-039/2015).

### Virus

The dengue virus serotype 2 (DENV2), strain New Guinea C (NGC) (GenBank M29095), was used for mouse infection. The New Guinea C strain was originally isolated from one infected patient followed by at least 38 mouse brain passages for neuroadaptation^34,60^. The virus stock used in the present work was obtained after eight more passages in the brain of Swiss newborn mice and subsequently propagated in Vero cells cultured in medium 199 with Earle salts (E199, Sigma), buffered with sodium bicarbonate and supplemented with 10% fetal bovine serum (FBS, Invitrogen). The supernatant of the infected cell culture was aliquoted and stored at −70 °C until use. Supernatant obtained from non-infected cell culture was used as mock controls.

### Infection of BALB/c mice with DENV2 by the intracerebral route

BALB/c mice were inoculated by the intracerebral (i.c.) route with 2,04 log_10_ PFU in 30 μL (40 LD_50_) of DENV2 diluted in E199 medium, which corresponds to 6 log_10_ virus RNA copies/mL. For evaluation of the kinetics of virus infection measured by tissue damages, animals (n = 5) were sacrificed 1, 3, 5 and 7 days post infection (dpi) and also on endpoints after the appearance of morbidity (from 8 to 11 dpi). Other experiments were conducted by monitoring mortality and morbidity for 13 days after infection (n = 10). Morbidity was expressed mainly by the appearance of hind leg paralysis and alterations in spinal column, by using a scale from 0 to 4 (0 = absence of morbidity, 1 = mild paralyses in one hind leg or alteration of the spinal column with a small hump, 2 = severe paralyses in one hind leg and alteration of the spinal column with a small hump or severe paralyses in both hind legs, 3 = severe paralyses in both hind legs and deformed spinal column, 4 = death). Control groups (n = 5) were inoculated with 30 μL mock and sacrificed at 3 and 7 days after inoculation. Before i.c. inoculation, animals were anesthetized with a mixture of ketamine-xylazine (100 mg/kg Ketamine; 10 mg/kg Xylazine) and for euthanasia they were overexposed to this mixture^61^.

### Dye inoculation in mouse brains

Animals (n = 3) were i.c. injected with 30 μL of Nankin dye, diluted 1:10 (v/v) in water, in the same position as virus inoculations, i.e., to the left of the sagittal suture through the coronal suture, between the parietal and frontal bones of mouse skull. For control, mice (n = 2) were injected with 30 μL of phosphate buffer saline (PBS) in the same position. The brains and cerebellums were collected from animals 5 minutes after injection for observation and photographic register of dye spread through the nervous tissues.

### Histological analysis

The brain and cerebellum samples from BALB/c mice infected with the DENV2 NGC or injected with mock were cleaved, fixed in buffered formalin (10%) and blocked in paraffin resin. The tissue sections, cut in 4 µm thick, were deparaffinized in xylene and rehydrated with decreasing concentrations of ethanol, as described elsewhere^62^. After washing in running water, the tissue sections were stained with hematoxylin and eosin for histological examination and visualized under Nikon ECLIPSE E600 microscope. Quantification of tissue lesions were performed by a semi quantitative analysis in brain and cerebellum sections stained with H.E. For each of the parameters used in the quantification of damages (hemorrhage, perivascular infiltrates and infiltrates in the meninges), a numerical scale ranging from 0 to 4 according to the severity and the extent of damage was assigned (0 = none, 1 = mild, 2 = moderate, 3 = severe and focally, 4 = severe and diffuse). Values were measured covering the entire histological slide, visualized by light microscopy (epifluorescence microscope NIKON ECLIPSE E600).

### Immunohistochemistry in the nervous tissues of BALB/c mice infected with DENV2

Paraffin-embedded cerebral and cerebellar tissues were cut in 4 µm thick sections, incubated 1 h at 60 °C, deparaffinized in xylene, hydrated in decreasing concentrations of ethanol and rinsed in distilled water. Subsequently, antigen retrieval of the tissue sections was performed in citrate buffer (Diagnostic Biosystems), pH 6.0, by pressure cooker at 100 °C for 5 min. Next, slides were rinsed with distilled water and washed with Tris-HCl buffer (3.8 mL of 1 N HCl, 0.6 g of Trizma® base, 8 g of NaCl and 1 L of distilled H_2_O, pH 7.4), blocked for endogenous peroxidase with 3% hydrogen peroxidase in methanol for 10 min and washed again with Tris-HCl buffer. Tissue sections were further blocked by incubation with protein block solution (Spring Bioscience) and washed with Tris-HCl buffer. The tissue sections were incubated overnight at 4 °C with anti-GFAP (1:200 dilution, Sigma) or anti–IBA-1 (1:200 dilution, Wako) antibodies for detection of astrocytes and microglia, respectively. Detection of DENV2 antigens was performed using an anti-NS3 polyclonal serum (1:50 dilution), produced in-house by inoculation of purified recombinant DENV2 NS3 protein in rabbit (kindly given by Dr. Ronaldo Mohana-Borges, Federal University of Rio de Janeiro, Brazil). The next day, slides were washed with Tris-HCl buffer, incubated with anti-rabbit antibody conjugated to horseradish peroxidase for 10 min and its complement for 15 min in a humid chamber at room temperature (REVEAL Biotin-free Polyvalent DAB Detection System, Spring Bioscience). Negative control samples were incubated only with the HRP-conjugated antibody. Slides were washed with Tris-HCl buffer and distilled water, revealed with diaminobenzidine (DAB Kit, Diagnostic Biosystems) and counterstained with Harris hematoxylin (Sigma). The material was analyzed by epifluorescence microscope NIKON ECLIPSE E600 with a Cool SNAP Prof COLOR camera attached.

### RNA isolation from the brain and cerebellum of DENV2 infected mice and the RT-qPCR assay

In order to quantify viral load, the brain and cerebellum samples obtained from mice inoculated with DENV2 and collected on days 1, 3, 5, 7 and 9–11 dpi, or from mock negative controls, were stored in liquid nitrogen until use. Half of the brain and cerebellum from each animal was polled and macerated in E199 medium for RNA isolation. Virus RNA extraction was performed using RNeasy Mini Kit (Qiagen) according to the manufacturer’s instructions. The RNA extracted from 75 μL of macerated tissue, corresponding to 30 mg of the organ, was eluted in 40 μL of sterile H_2_O. DENV2 NGC sample isolated from infected Vero cells or a pool of newborn mouse brains inoculated with DENV2 NGC were used as positive controls of RNA extraction.

Isolated RNAs were *in vitro* reversely transcribed (RT) using the High Capacity cDNA Reverse Transcription Kit (Applied Biosystems). Reactions were carried out with 20 μl of RNA from each sample added to 20 μl of the master mix and random primers provided in the kit. Amplification conditions were: 25 °C for 10 min, 37 °C for 120 min and 85 °C for 5 min. The resulting cDNA was stored at −20 °C and used for qPCR on the next day. The quantitative PCR assays were performed using TaqMan® One-Step PCR kit (Applied Biosystem). Each reaction was set up with a volume of 20 μL containing master mix kit, probe (10 μM), primers (0.1 μM), H_2_0 and 5 μL of cDNA template. For each sample, amplification occurred in duplicate in a 96-well reaction plate, including negative and positive controls of RNA extraction, as well the negative (no template control) and positive (with recombinant plasmid containing the target sequence) PCR controls. Amplification reactions were performed by ABI Prism 7500 (Applied Biosystems) at Laboratory of Virological Technology (LATEV, BioManguinhos/FIOCRUZ). The qPCR conditions were: 95 °C for 10 min, 45 cycles of 95 °C for 15 s, followed by 60 °C for 1 min and 1 min at 72 °C.

The target sequence selected for PCR amplification was a specific conserved region of 73 bp in the DENV2 envelop gene sequence, at position 1570 to 1642 of the DENV2 NGC genome (Gen Bank number: M29095): TGGTTCCTAGACCTGCCGTTACCATGGCTACCCGGAGCGGACACACAAGGATCAAATTGGATACAGAAAGAGA. Primers used for amplification were: forward 5′-TGGTTCCTAGACCTGCCGTTA-3′, Reverse 5′-TCTCT TTCTGTATCCAATT TGAT CCTT-3′ and fluorogenic probe 5′-FAM-CATGGCTACCCGGAGCGGACAC–TAMRA-3′. The standard curve used in qPCR assays was generated from a serial dilution of a recombinant plasmid containing the target sequence. Briefly, for construction of this plasmid, the 73 bp DENV2 NGC fragment from the cDNA template was cloned into the TOPO TA plasmid backbone (TOPO® TA Cloning® Kits, Thermo Fisher Scientific), resulting in a 4004 bp plasmid. *Escherichia coli* Top 10 strain was transformed with 1 ng of the recombinant plasmid. The plasmid DNA was purified using Qiagen Plasmid Maxi Kit (Qiagen) and quantified by nanodrop. Copy numbers were determined by Avogadro formula (1010 copies/µL). The standard curve was plotted as a tenfold serial dilution 10^7^ to 10^1^ copies/µL and used to quantify tested samples.

### DENV2 infection in primary culture of neuronal cells

Dengue infection was evaluated in primary culture of glial and neuronal cells (mixed cell culture) through the detection of viral antigens by immunofluorescence. Primary cells were obtained from the cerebral cortex of two newborn BALB/c mice brains. The cerebral cortex was separated from the brain in PBS aqueous surface and cells were dissociated in Dulbecco’s Modified Eagle Medium: Nutrient mixture F12 (DMEM-F12, Sigma) supplemented with 33 mM glucose, 2 mM glutamine, 3 mM sodium bicarbonate, 0.5 mg/ ml of penicillin/streptomycin, 2.5 g/mL of fungizone and 10% FBS (v/v). After 15 min of debris settling, the supernatant was centrifuged at 1500 rpm for 2 min and the pellet containing primary mixed cells was suspended with the same medium.

For DENV2 detection, 3.0 × 10^4^ cells/well were cultured in sterile chamber slides (Lab-Tek, Nunc), pre-coated with poly-L-lysine (5 g/mL), at 37 °C in a humidified 5% CO_2_ and 95% air atmosphere. After 7 days of culturing, the medium was replaced and after 3 more days cells were infected with DENV2. For virus infection, monolayers were washed with BSS (8 g/L NaCl, 0.4 g/L KCL, 0.012 g/L CaCl_2,_ 0.154 g/L MgSO_4_.7H_2_0, 0.39 g/L Na_2_HPO_4_.12H_2_0, 0.15 g/L KH_2_PO_4_, 1.1 glucose, 0.0025 g/L phenol red) without FBS (pH 7.4) and 6 × 10^4^ PFU of DENV2 (MOI 2) were added to each well. Cells were incubated at 37 °C and 5% CO_2_ for 1 h. Subsequently, the infectious material was discarded, modified DMEM-F12 medium was added and cells were maintained at 37 °C and 5% CO_2_ for 48 h.

### Immunofluorescence of primary cultured of neuronal cells infected with DENV2

After virus infection, cell monolayers were washed with BSS and fixed with 4% paraformaldehyde (pH 7.4) for 10 min at room temperature. Cells were then washed with 0.1 M phosphate buffer (pH 7.4), permeabilized with saponin (0.1 M phosphate buffer, 1% BSA, 0.6% saponin) for 10 min, washed again and incubated with the blocking buffer (0.1 M phosphate buffer, 1% BSA, 0.2% saponin) for 15 min. Next, cell monolayers were incubated for 1 h with 150 μL/well of specific antibodies, diluted with 0.1 M phosphate buffer with 0.1% BSA and 0.2% saponin, at 37 °C in a humid dark chamber. Detection of DENV2 antigens was performed using an anti-NS3 polyclonal serum, raised in rabbit inoculated with the recombinant DENV2 NS3 protein, or anti-envelope (E) antibodies obtained from rabbit immunized with a DNA vaccine encoding the ectodomain of the DENV2 E protein (pE1D2)^63^. Subsequently, cells were washed with 0.1 M phosphate buffer and incubated for 1 h with anti-rabbit antibody (150 µL/well) conjugated with FITC (1:100 dilution, Southerm Biotechnology), at 37 °C in a humid dark chamber. Anti-GFAP antibody conjugated with TRITC fluorophore was also used for detection of astrocytes (1:200 dilution, Sigma). Slides were mounted with Vectashield medium (Vector Laboratories Inc., USA) and analyzed by fluorescence confocal microscope (Olympus FV10i-O).

### Statistical analysis

Statistical analyses were performed with GraphPad prism software version 6. Statistical significance was evaluated using the non-parametric Mann-Whitney test. Values were considered significant at p < 0.05.
